# The Multi-Dimensional Action Map of Resveratrol Against Alzheimer’s Disease: Mechanism Integration and Treatment Strategy Optimization

**DOI:** 10.3390/nu17213451

**Published:** 2025-10-31

**Authors:** Yichen Liu, Yadan Dong, Zhen Cao, Yixuan Ji, Xiaoxin Cheng, Xu Zheng

**Affiliations:** College of Basic Medical Science, Dalian Medical University, Dalian 116044, China; liuyichen1686@163.com (Y.L.); 15247442379@163.com (Y.D.); caozhen@stu.glmc.edu.cn (Z.C.); 15045015767@163.com (Y.J.)

**Keywords:** Alzheimer’s disease, resveratrol, anti-inflammatory, neuroprotection, mitochondrial homeostasis

## Abstract

Alzheimer’s disease (AD) represents a prevalent neurodegenerative disorder marked by a gradual decline in cognitive and behavioral functions. Despite advancements in elucidating several potential mechanisms underlying the pathogenesis of AD, there remains a limitation in effective supplements or medications for its intervention. Resveratrol, a natural antioxidant, has emerged as a significant player in the treatment of AD. This article reviews the role of resveratrol in four key aspects: amyloid plaque deposition and neurofibrillary tangles, inflammatory response and oxidative stress, energy metabolism and mitochondrial homeostasis, and neuroprotection and regeneration. Furthermore, we also explore treatment strategies to enhance the therapeutic effect of resveratrol.

## 1. Introduction

Alzheimer’s disease (AD) is an age-related neurodegenerative disorder characterized by progressive cognitive dysfunction, primarily impacting the hippocampus and cerebral cortex. This condition progressively impairs memory and logical thinking abilities, ultimately culminating in disorders of consciousness [1]. Despite extensive research into the multifaceted and complex nature of this syndrome in recent years, the precise molecular mechanisms underlying AD remain incompletely understood. The etiology of AD may be attributed to a complex interplay of various factors, including senile plaques (SPs), neurofibrillary tangles (NFTs), neuronal loss, chronic neuroinflammation, oxidative stress, and inadequate energy supply [2]. The absence of drugs capable of reversing or halting the progression of AD poses a significant public health challenge. Several medications have been approved to relieve the symptoms of AD, such as cholinesterase inhibitors and N-Methyl-D-Aspartate (NMDA) noncompetitive antagonists, but the complex mechanisms involved suggest that these compounds yield a range of side effects with only modest benefits [3].

Resveratrol, a natural compound present in grapes, red wine, peanuts, and berries, has been recognized for its benefits as a dietary supplement in the prevention of cardiovascular, brain, metabolic diseases, and cancer [4]. Recently, researchers have increasingly focused on the potential of resveratrol in slowing down the aging process and in the prevention of neurodegenerative diseases. It has been reported that resveratrol can activate sirtuin 1 (SIRT1), leading to an extension of lifespan in various model organisms, including Saccharomyces cerevisiae, Caenorhabditis elegans, Drosophila melanogaster, and short-lived seasonal insects [5]. Mammalian experimental studies have demonstrated that resveratrol activates both SIRT1 and AMPK signaling, which delays aging, extends lifespan, and improves cognitive deficits [6]. More significantly, resveratrol is a natural small-molecule substance that can cross the blood–brain barrier (BBB), has low toxicity and few side effects, and has good safety and tolerance [7]. The therapeutic and preventive efficacy of resveratrol in AD has been supported by phase II clinical trials involving patients with mild to moderate AD. The findings suggest that resveratrol is detectable in cerebrospinal fluid, is safe and well tolerated, and exerts a positive influence on AD biomarker trajectories [8]. In this review, we explore the therapeutic effects of resveratrol on AD in various aspects and its intrinsic molecular mechanisms, as well as the future potential of new routes of administration for the treatment of AD.

## 2. Mechanisms of Resveratrol in AD

Resveratrol slows down the progression of AD through a multi-target, multi-pathway synergism. Its core mechanisms can be summarized in four interlocking actions: clearance of amyloid plaques and NFTs, suppression of neuroinflammation and oxidative stress, restoration of energy metabolism and mitochondrial homeostasis, and promotion of neuroprotection and regeneration. By integrating “toxic-protein removal, inflammation inhibition, energy recovery, and neural regeneration,” resveratrol offers a promising multi-target therapeutic strategy for AD intervention.

### 2.1. Effect of Resveratrol on Amyloid Plaque Deposition and Neurofibrillary Tangles in AD

**Figure 1:**
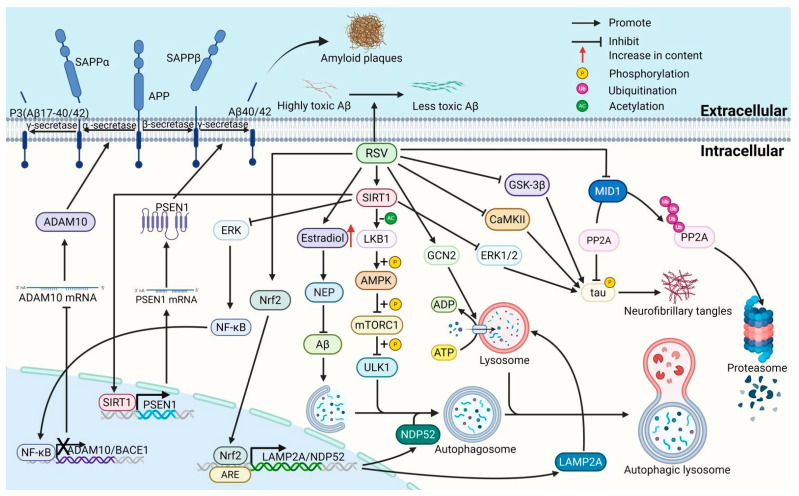
The mechanisms of resveratrol inhibit amyloid plaque deposition and NFTs. (1) RSV reduces the production of Aβ and promotes the degradation of Aβ through affecting the expression of ADAM10, BACE1, PSEN1 and NEP. (2) RSV reduces and attenuates Aβ neurotoxicity by inhibiting the aggregation of Aβ from low molecular weight oligomers to high molecular weight oligomers. (3) RSV regulates the permeability of the BBB by affecting the levels of LRP-1 and TTR, thereby promoting the transport and clearance of Aβ. (4) RSV promotes autophagic degradation of Aβ by activating the SIRT1/LKB1/AMPK/mTORC1/ULK1 pathway and upregulating proteins associated with the autophagic-lysosomal pathway, including NDP52, LAMP2A and GCN2. (5) RSV inhibits tau protein hyperphosphorylation through PI3K/AKT/GSK-3β, CaMKII and ERK1/2 pathways. Resveratrol also promotes tau dephosphorylation by inhibiting MID1-mediated ubiquitination degradation of PP2A. sAPPα/β, soluble amyloid precursor protein α/β; APP, amyloid β precursor protein; P3 (Aβ17-40/42), APP non-amyloidogenic fragment p3 (Aβ17-40/42); PSEN1, presenilin 1; ADAM10, a disintegrin and metalloproteinase domain-containing protein 10; BACE1, β-secretase 1; NF-κB, nuclear factor kappa-light-chain-enhancer of activated B Cells; SIRT1, sirtuin 1; Nrf2, nuclear factor erythroid 2-related factor 2; NEP, neprilysin; ERK1/2, extracellular signal-regulated kinase1/2; NDP52, nuclear dot protein 52 kDa; LAMP2A, lysosomal-associated membrane protein 2A; LKB1, liver kinase B1; AMPK, AMP-activated protein kinase; mTORC1, mechanistic target of rapamycin complex1; ULK1, unc-51 like autophagy activating kinase 1; ATP, adenosine triphosphate; ADP, adenosine diphosphate; GCN2, general control nonderepressible 2; CaMKII, calmodulin-dependent protein kinase II CaMKII; GSK-3β, glycogen synthase kinase-3β; tau, tubulin-associated unit; PP2A, protein phosphatase 2A; MID1, midline 1.

The initial pathological characteristics of AD consist of the deposition of amyloid plaques and the formation of NFTs, which exert significant toxic impacts on neurons. These pathological features may also serve as the primary factors contributing to synaptic loss, neuroinflammation, and the gradual decline in cognitive function associated with AD. Resveratrol does not act through a single pathway; instead, it “one-clicks, multi-targets” the two core pathologies of AD: amyloid plaque deposition and NFT formation (Figure 1).

#### 2.1.1. Resveratrol Reduces Amyloid Plaque Deposition by Modulating Enzymes Involved in Aβ Production

The formation of amyloid plaques results primarily from the aberrant deposition of amyloid β-protein (Aβ). Aβ is a polypeptide containing 39 to 43 amino acids produced by the proteolysis of amyloid precursor protein (APP) by β- and γ-secretase. APP is a transmembrane protein expressed predominantly at the synapses of neurons, and the Aβ fragment is located within its transmembrane region [9]. APP can be cleaved directly by α-secretase, which is further cleaved by γ-secretase to form P3 (Aβ17-40/42), a process that does not produce Aβ. In the alternative way, APP can also be cleaved by β-site APP cleavage enzyme (BACE1) and release the extracellular domain of the soluble APP beta protein (sAPPβ), after which the APP *C*-terminal fragment is cleaved by the γ-secretase complex at one of several sites within the range of 40 to 44, generating the neurotoxic Aβ peptide [10]. Therefore, inhibiting the activity of β- and γ-secretases may be a key target for AD drug treatment, while enhancing the activity of α-secretases may also offer a comparable therapeutic effect against AD.

γ-Secretase is a multiprotein complex consisting of Presenilin1 (PSEN1), Nicastrin, Anterior pharynx defective-1 (Aph-1) and Presenilin enhancer-2 (Pen-2), all four of which are necessary for full proteolytic activity [11]. Research has demonstrated that resveratrol specifically upregulates PSEN1 expression through SIRT1-mediated binding to the PSEN1 gene locus, while showing no significant effect on the expression of other γ-secretase components [12]. However, the study did not extend its investigation to determine whether resveratrol-mediated PSEN1 upregulation could modulate γ-secretase enzymatic activity and influence Aβ generation.

Resveratrol also modulates BACE1 activity through interacting with the transcription factor nuclear factor-κB (NF-κB). NF-κB, a pivotal transcription factor involved in inflammation, oxidative stress, and apoptosis regulation, has been identified as a positive regulator of BACE1 expression. Researchers further identified two functional NF-κB-binding elements in the BACE1 promoter region, and both knockdown of NF-κB p65 and treatment with non-steroidal anti-inflammatory drugs (NSAIDs) inhibited NF-κB-induced BACE1 transcriptional activation [13]. Resveratrol can inhibit the NF-κB pathway, but without the significant side effects of non-steroidal anti-inflammatory drugs (NSAIDs), reflecting the great potential to reduce the production of Aβ [14,15]. However, the ADAM10 promoter also contains an NF-κB binding site. Under oxidative stress in the brain, inhibition of the SIRT1/ERK/NF-κB axis downregulates ADAM10 expression, which switches APP production from non-amyloid to amyloid. Resveratrol counteracts this process by activating the SIRT1/ERK/NF-κB axis, thereby upregulating ADAM10 transcription and reducing amyloid production [16]. However, resveratrol can inhibit NF-κB activation, which seems to be contradictory. This complexity may reflect a context-dependent modulation of the NF-κB pathway, and the underlying molecular biological mechanisms require further investigation.

#### 2.1.2. Resveratrol Reduces Amyloid Plaque Deposition by Neprilysin (NEP) Involved in Aβ Clearance

The deposition of amyloid plaques is not only affected by the above-mentioned Aβ-producing enzymes, but also by enzymes related to amyloid plaque degradation and Aβ clearance. NEP is recognized as a key amyloid-degrading enzyme and critically contributes to the degradation of Aβ. This enzyme is also an important neuropeptidase that regulates brain function by controlling the catabolism of sensory and inflammatory neuropeptides, including tachykinins and neurokinins. Studies have shown that NEP protein and mRNA levels in the mouse cerebral cortex decrease with age, independent of transgene status [17]. Resveratrol can increase the level of NEP by increasing the level of estradiol, and further reduce Aβ deposition [18].

#### 2.1.3. Resveratrol Reduces Amyloid Plaque Deposition by Modulating BBB Permeability

The BBB is critically involved in maintaining the Aβ homeostasis and amyloid plaque clearance in the central nervous system. BBB dysfunction, particularly decreased levels of low-density lipoprotein receptor-associated protein 1 (LRP1) and elevated levels of receptor for advanced glycation end-products (RAGE) in the BBB, impairs the transport of Aβ from the brain across the BBB into the peripheral circulation [19]. RAGE serves as the primary receptor for Aβ influx at the BBB, mediating the delivery of Aβ from the blood into the brain [20]. RSV can protect the integrity of the BBB by increasing Claudin-5 and reducing the RAGE and matrix metalloproteinase-9 (MMP-9) [21]. LRP1 exists in two forms: membrane-bound and soluble. The membrane-bound form of LRP1 is mainly expressed on the near luminal side of the BBB and mediates Aβ crossing the BBB into the circulation, thereby clearing it from the brain. The other is a soluble form of LRP1 (sLRP1), which can bind to free plasma Aβ in the peripheral circulation to form sLRP1-Aβ complex, which is cleared by the kidney and liver [22]. However, under oxidative stress conditions, membrane-bound LRP1 is oxidized and cannot bind to Aβ, which increases Aβ deposition in the hippocampus of AD patients’ brains. Consistent with this, brain clearance of Aβ(1–40) across the BBB is reduced in AD patients and animal models due to Aβ self-aggregation and the formation of LRP1 ligand complexes [23]. Transthyretin (TTR) binds to Aβ peptides, preventing their aggregation and toxicity, while also enhancing the levels of low-density LRP1 to facilitate Aβ internalization and degradation [24]. Resveratrol treatment increased TTR and low-density LRP1 levels, thereby reducing Aβ deposition in AD models. Notably, TTR was elevated in resveratrol-treated mice, but hepatic TTR gene transcription remained unchanged, suggesting that resveratrol enhances TTR stability rather than TTR transcription [25].

#### 2.1.4. Resveratrol Reduces Amyloid Plaque Deposition by Modulating Protein-Degradation Systems

The clearance of misfolded or abnormal proteins in AD relies heavily on the cellular degradation system. This system comprises three primary mechanisms: the ubiquitin-proteasome system (UPS), the autophagy-lysosomal pathway (ALP), and the coordinated interaction of molecular chaperones with UPS or ALP. Accumulating evidence suggests that impaired lysosomal acidification in AD patients is a significant contributor to the abnormal deposition of proteins [26]. PSEN1 is required for lysosomal proteolysis and autophagy. However, Alzheimer’s disease-associated PS1 mutation disrupts the targeting of the V0a1 subunit of v-ATPase to lysosomes, resulting in lysosomal acidification disorder [27]. In autophagy-disordered cells, the expression of PSEN1 is increased and γ-secretase activity is enhanced, leading to increased Aβ synthesis. Resveratrol, as an autophagy inducer, can reverse this phenomenon through the general control of nonderepressible 2 (GCN2) [28]. The transcription factor Nrf2 has also been identified as a key regulator of autophagy. Nrf2-deficient mice showed more intracellular aggregation of misfolded proteins and reduced levels of Sequestosome 1 (SQSTM1/p62), nuclear dot protein 52 (CALCOCO2/NDP52), serine/threonine-protein kinase 1 (ULK1), autophagy-related protein 5 (ATG5), and gamma-aminobutyric acid receptor-associated protein-like 1 (GABARAPL1) in neurons. Nrf2 can promote chaperonate-mediated autophagy (CMA) by increasing the LAMP2A level in lysosomes [29]. Furthermore, Nrf2 induces the expression of the autophagy aptamer NDP52, which contains three antioxidant response elements (ARE) in its promoter region [30].

Resveratrol has been reported to elevate intracellular calcium levels, thereby stimulating AMPK activation through the calcium/calmodulin-dependent protein kinase kinase-β (Ca^2+^/CaMKKβ) pathway. Subsequently, AMPK inhibits mechanistic target of rapamycin (mTOR), leading to the induction of autophagy and lysosomal degradation of Aβ. Oral administration of resveratrol was detected in the brains of APP/PS1 transgenic mice, where it activated AMPK and reduced brain Aβ levels and cortical deposition [31]. However, conflicting evidence suggests that neither CaMKKβ nor SIRT1 inhibition affects resveratrol-mediated AMPK activation. Treatment of Neuro2a cells with 10 μM resveratrol resulted in a decrease in the AMP: ATP ratio, indicating that resveratrol must stimulate AMPK activation in Neuro2a cells through mechanisms independent of AMP elevation. Furthermore, resveratrol-induced AMPK activation in cortical and dorsal root ganglion sensory neurons requires liver kinase B1 (LKB1), but not SIRT1 [32]. These contradictory findings may be explained by the dose-dependent effect of resveratrol. At a concentration of 25 μM, resveratrol activates SIRT1 by increasing the level of NAD^+^, which deacetylates LKB1 and further activates AMPK. This AMPK activation, in turn, indirectly elevates NAD^+^ levels, creating a positive feedback loop. In contrast, at a concentration of 50 μM, resveratrol inhibits phosphodiesterase (PDE), leading to increased cAMP levels and elevated intracellular calcium, thereby stimulating AMPK phosphorylation via CaMKKβ [33]. Emerging evidence reveals a paradoxical relationship between mTORC1 and AMPK signaling in amyloid-β (Aβ) regulation. Although inhibition of mTOR complex 1 (mTORC1) promotes autophagy-mediated Aβ clearance, both pharmacological activation and genetic overexpression of AMPK unexpectedly fail to enhance autophagic activity in neurons and astrocytes. Intriguingly, AMPK activation appears to paradoxically increase Aβ secretion, suggesting distinct regulatory mechanisms in amyloid processing between these two pathways [34,35]. A 20 μM concentration of resveratrol appears to cause Aβ production in a Swedish APP (APPsw) stable-expressing cell mutant by stabilizing APP and activating AMPK-mediated inhibition of trypsin-like proteasome activity. In contrast, 50 μM concentration of resveratrol decreased Aβ secretion and β-secretase activity during any treatment period [36]. This may explain why resveratrol appears to have two opposing effects on Aβ clearance.

#### 2.1.5. Resveratrol Reduces Amyloid Plaque Deposition by Diminishing Aβ Protein Aggregation

Resveratrol not only contributes to Aβ homeostasis but also inhibits the aggregation of Aβ from lower molecular weight oligomers into higher molecular weight oligomers. It disrupts preformed Aβ aggregates, thereby reducing their cytotoxicity. Resveratrol can directly bind to Aβ(1–42) and inhibit fibril Aβ(1–42) formation and its associated cytotoxicity in a dose-dependent manner. However, it could not prevent Aβ(1–42) oligomerization leading to the formation of numerous Aβ(1–42) oligomers [37]. The binding affinity of resveratrol to fAβ(1–42) is higher than to monomeric Aβ(1–42), whereas its binding to fAβ(1–40) is weaker compared to monomeric Aβ(1–40) [38]. Resveratrol also selectively remodels three conformational isomers (soluble oligomers, fibrillar intermediates, and amyloid fibrils) into alternative aggregates that are nontoxic, high-molecular-weight, and unstructured [39]. This may explain why resveratrol protects SK-N-BE cells from Aβ(1–42) toxicity in a SIRT1-independent manner [40].

#### 2.1.6. Resveratrol Suppresses Neurofibrillary Tangles (NFTs) by Inhibiting the Hyperphosphorylation of Tau Protein

Tau protein is essential for microtubule assembly, stabilization, and regulation, which are critical for neuronal and brain function. Tau hyperphosphorylation triggers its aggregation into paired helical filaments (PHFs) and NFTs, resulting in serious consequences such as physiological function impairment, apoptosis and neuronal loss [41]. Resveratrol, a specific SIRT1 activator, effectively counteracts the reduction in SIRT1 activity, ERK1/2 phosphorylation, tau hyperphosphorylation and cognitive impairment induced by lateral intraventricular injection of streptozotocin (ICV-STZ) in rats [42]. Furthermore, resveratrol activates SIRT1 to deacetylate splicing factor 9G8 at Lys24, suppressing tau exon 10 exclusion and improving learning and spatial memory in Htau mice [43]. Protein phosphatase 2A (PP2A) is the primary phosphatase for tau dephosphorylation. Resveratrol significantly enhances the activity of PP2A, thereby diminishing tau phosphorylation at PP2A-dependent epitopes. The investigators further found that the enhanced PP2A activity results from resveratrol-mediated downregulation of MID1 ubiquitin ligase, which normally promotes ubiquitination and subsequent degradation of PP2A catalytic subunits following microtubule association. Notably, MID1 expression is elevated in AD tissues, suggesting that resveratrol may be a potential drug to interfere with MID1-PP2A complex-induced tau dephosphorylation and reduce the formation of PHFS and NFTS in AD patients [44]. Additionally, resveratrol reduces tau hyperphosphorylation and NFTs by inhibiting glycogen synthase kinase (GSK-3β) and calmodulin-dependent protein kinase II (CaMKII), both of which are critical kinases for tau hyperphosphorylation [45].

### 2.2. Effects of Resveratrol on Neuroinflammation and Oxidative Stress in AD

**Figure 2:**
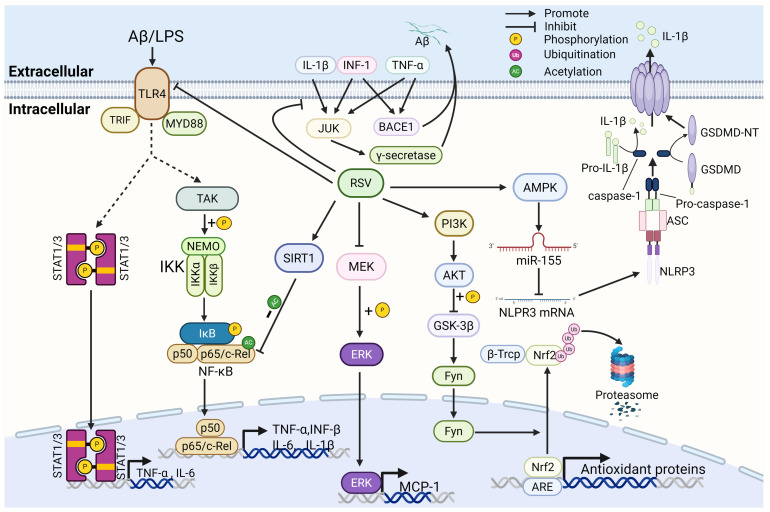
The mechanisms of resveratrol inhibit neuroinflammation and oxidative stress. (1) RSV suppressed the production of BACE1 and γ-secretase by reducing inflammatory factors, thereby diminishing Aβ generation. (2) RSV attenuates glutamate-induced MEK/ERK activation and reduces MCP-1 expression. (3) RSV inhibits TNF-α and IL-6 production via suppression of the TLR4/NF-κB and STAT1/3 pathways. (4) RSV mediates deacetylation of RelA/p65 through SIRT1 activation, thereby blunting downstream inflammatory signaling. (5) RSV inhibits pyroptosis through AMPK/miR-155/NLRP3 pathways. (6) RSV promotes the expression of antioxidant proteins by activating the PI3K/AKT/GSK-3β/Nrf2 pathway. RSV, resveratrol; LPS, lipopolysaccharide; STAT1/3, signal transducer and activator of transcription1/3; TLR4, toll-like receptor 4; MYD88, myeloid differentiation primary response 88; TRIF, TIR-domain-containing adapter-inducing interferon-β; TAK, TGF-β-activated kinase 1; NEMO, NF-κB essential modulator; IKKα/β, inhibitor of nuclear factor kappa B kinase subunit α/β; IκB, inhibitor of kappa B; p50, nuclear factor kappa B subunit p50; p65, nuclear factor kappa B subunit p65; SIRT1, sirtuin 1, MEK, MAPK/ERK kinase; ERK, extracellular signal-regulated kinase; MCP-1, monocyte chemoattractant protein-1; PI3K, phosphoinositide 3-kinase; AKT, protein kinase B; GSK-3β, glycogen synthase kinase-3β; Fyn, proto-oncogene tyrosine-protein kinase Fyn; Nrf2, nuclear factor erythroid 2-related factor 2;β-TrCP, β-transducin repeat-containing protein; NLRP3, NLR family pyrin domain containing 3; AMPK, AMP-activated protein kinase; JUK, *c*-Jun *N*-terminal kinase; BACE1, β-Secretase 1; Caspase-1, cysteine-dependent aspartate-specific protease 1; ASC, apoptosis-associated speck-like protein containing a CARD; GSDMD, Gasdermin D.

Numerous studies have established a strong association between AD and neuroinflammation and oxidative stress. In addition to the accumulation of β-amyloid plaques, hyperphosphorylation of tau protein and NFTS, the pathology of AD is often accompanied by astrogliosis and microglial activation, which trigger a number of proinflammatory and anti-inflammatory mediators [46]. For example, activation of microglia releases large amounts of ROS, hydroxyl radicals, superoxide and peroxide radicals, hydroxyl peroxides, which induce protein oxidation, lipid peroxidation and DNA fragmentation, ultimately leading to neuronal inflammation and cell death [47]. Microglia activation and astrogliosis are generally considered secondary to neurodegeneration, but recent studies have revealed that abnormal inflammatory signaling and glial activation may already occur early in AD pathogenesis [48]. Alleviating neuroinflammation has become a key therapeutic target for resveratrol in the treatment of AD (Figure 2).

#### 2.2.1. Resveratrol Reduces Amyloid Plaque Deposition by Down-Regulating Inflammatory Cytokines

Some inflammatory cytokines such as interferon-γ (IFN-γ), interleukin-1β (IL-1β) and tumor necrosis factor-α (TNF-α) can specifically stimulate neuronal γ-secretase activity through Jun N-terminal kinase (JNK) and mitogen-activated protein kinase (MAPK) pathways, leading to increased Aβ production [49]. Moreover, TNF-α and IFN-γ could increase the levels of endogenous BACE1, APP and Aβ in astrocytes. At the same time, given the abundance of astrocytes relative to neurons, activated astrocytes may serve as a significant source of Aβ during neuroinflammation in AD [50]. Aβ can also stimulate glial cells to secrete more inflammatory factors, which seems to form a positive feedback loop and gradually exacerbates disease progression [48]. In a retrospective clinical study, resveratrol significantly decreased MMP9 while increasing macrophage-derived chemokine (MDC), interleukin-4 (IL-4), and fibroblast growth factor-2 (FGF-2) in cerebrospinal fluid. It reduced neuroinflammation and induced adaptive immune responses. Although resveratrol did not alter total tau and *p*-tau content in CSF, it reduced Aβ(1–42) levels and slowed the progressive cognitive and functional decline in mild to moderate AD subjects [51].

#### 2.2.2. Resveratrol Suppresses Inflammation-Related Signaling Pathways and the Production of Inflammatory Mediators

MAPK plays an important role in inflammatory response, influencing LPS-induced gene expression through signal transducer and activator of transcription 1 (STAT1) phosphorylation and NF-κB nuclear translocation [52]. Therefore, inhibition of MAPK, which reduces inflammation and enhances neuroprotection, may be a potential target of resveratrol in the treatment of neuroinflammation in AD [53]. Resveratrol has been shown to inhibit LPS-induced MAPK activation and reduce the expression of inflammatory mediators such as nitric oxide (NO), prostaglandin E2 (PGE2), TNF-α and IL-1β through activation of the phosphatidylinositol 3-kinase (PI3K) pathway [54].

Glutamate, implicated in AD pathogenesis, upregulates chemokines such as monocyte chemoattractant protein-1 (MCP-1) and chemokine ligand 2 (CCL2). Resveratrol can downregulate glutamate-induced IL-1β expression in rat hippocampal slice cultures, while simultaneously reducing MCP-1 mRNA expression and protein release through MEK/ERK pathway inactivation [55]. However, one study reported that resveratrol did not alter LPS-stimulated ERK1/2 or p38 phosphorylation in either microglia or astrocytes, only marginally suppressed LPS-induced JNK phosphorylation in astrocytes, but significantly inhibited LPS-triggered NF-κB activation. Therefore, the suppression of LPS-induced proinflammatory cytokine expression and release by resveratrol may be primarily mediated by downstream signaling molecules of the MAPK pathway [56].

#### 2.2.3. Resveratrol Inhibits Astrocyte-Associated Inflammation

Astrocytes significantly contribute to neuroinflammation in AD. Upon exposure to harmful stimuli, astrocytes are induced and activated, releasing a variety of cytokines such as IFN-γ, IL-1β and TNF-α, which exacerbate neuroinflammation [57]. Glial fibrillary acidic protein (GFAP) is a neuroinflammatory marker, which is mainly expressed by activated astrocytes. Resveratrol can reverse LPS-induced astrocyte activation and GFAP expression by restoring LPS-inhibited SIRT1, and improve hippocampus-dependent spatial learning and memory in mice [58]. Moreover, resveratrol also inhibits gliosis activated by Aβ(1–42) in primary rat astrocytes, which is considered to be one of the most important markers of AD, through SIRT1 activation and SIRT2 inhibition [59]. Heme oxygenase-1 (HO-1) is essential for mediating the suppressive effects of resveratrol on LPS-induced upregulation of inflammatory mediators and enhanced nuclear translocation of NF-κB p65 in hippocampal astrocytes. Moreover, resveratrol elevated the levels of the anti-inflammatory cytokine IL-10, independent of LPS stimulation [53]. Compared to astrocyte cultures from neonatal rats, hippocampal astrocytes derived from adult and aged rats exhibited decreased antioxidant defenses and increased inflammatory responses. Resveratrol could enhance the neuroprotective effects by increasing glutathione (GSH) content and glutathione synthase activity while reducing TNF-α and IL-1β levels [60].

#### 2.2.4. Resveratrol Inhibits Microglia-Associated Inflammation

Microglia, the resident innate immune cells of the central nervous system, undergo activation upon sustained inflammatory stimulation, initiating a series of neuroinflammatory cascades. The proportion of activated microglia is much higher in advanced AD patients compared to controls. The marked increase in activated microglia and their role in inflammatory responses and neurotoxin secretion contribute to neuronal degeneration in AD [61].

The innate immune receptor Toll-like receptor 4 (TLR4), a member of the Toll-like receptor (TLR) family, is located on the surface of microglia. Upon binding to LPS, TLR4 initiates signal transduction and triggers TAK/IKK/NF-κB inflammatory pathways by engaging two distinct adaptor proteins: myeloid differentiation primary response 88 (MyD88) and TIR-domain-containing adapter-inducing interferon-β (TRIF) [62]. Gene profiling of the human brain revealed increased expression of TLR4, TNF-α, and IL-6 genes in the frontal cortex of AD patients postmortem compared with age-matched controls [63]. In addition, Aβ can trigger microglial activation by interacting with a variety of Toll-like receptors, including TLR4 [47,64]. Resveratrol attenuates LPS-mediated proinflammatory responses by disrupting TLR4 oligomerization and further suppresses STAT1/3 and NF-κB signaling. Furthermore, resveratrol dose-dependently inhibits fibrillar Aβ-triggered microglial activation, reducing STAT1, STAT3, and IκBα phosphorylation, as well as TNF-α and IL-6 secretion. Resveratrol reduced Aβ levels, amyloid deposition and activated microglia in the brains of APP/PS1 mice. It should be emphasized that this effect cannot be attributed solely to the anti-amyloidogenic activity of resveratrol, as a clear trend towards reduced numbers of activated microglia of the same plaque size was observed in resveratrol-treated mice compared with untreated controls [15]. Aging and Aβ oligomers can increase TLR4 expression on neuronal surfaces and enhance LPS/TLR4-mediated intracellular Ca^2+^ increase-mediated apoptosis [65]. Resveratrol may act as a natural TLR4 antagonist, like CAY10614 (TLR4 antagonist), to reverse this effect [66]. Aβ stimulation leads to acetylation of RelA/p65 at lysine 310 in microglia, which amplifies the NF-κB pathway and inflammatory responses. However, resveratrol can activate SIRT1 and deacetylate RelA/p65, which has a strong neuroprotective effect [67].

The NLRP3 inflammasome, a critical component of the innate immune system, is triggered by pathogen-associated molecular patterns (PAMPs) or damage-associated molecular patterns (DAMPs). Upon activation, NLRP3 oligomerizes with apoptosis-associated speck-like protein containing a CARD (ASC) and pro-caspase-1, forming an inflammasome complex. The inflammasome complex mediates the proteolytic cleavage of IL-1β and IL-18 through caspase-1 activation, facilitating their release into the extracellular space. Concurrently, activated caspase-1 catalyzes the N-terminal cleavage of Gasdermin D (GSDMD), generating membrane pores that enable IL-1β and IL-18 secretion while permitting water influx, ultimately triggering pyroptotic cell death [68]. By activating AMPK to downregulate LPS-induced miR-155, resveratrol inhibits the formation of the NLRP3 inflammasome and the production of IL-1β and GSDMD-NT, thereby ultimately attenuating NLRP3 inflammasome activation and pyroptosis in microglia [69]. Notably, resveratrol suppresses Aβ-induced microglial activation, as well as caspase-1 and IL-1β overexpression via the thioredoxin-interacting protein (TXNIP)/thioredoxin (TRX)/NLRP3 signaling pathway. This further supports its role in modulating NLRP3 inflammasome-mediated neuroinflammation in AD [70].

#### 2.2.5. Resveratrol Inhibits Oxidative Stress-Related Pathways

Brain tissue is particularly vulnerable to oxidative stress owing to its elevated oxygen demand, rich content of polyunsaturated fatty acids, and relatively low intrinsic antioxidant defense capacity [71]. Growing evidence indicates that the reduced expression and activity of antioxidant proteins along with subsequent oxidative stress are the underlying causes of brain aging processes and neurodegenerative diseases [72]. As a natural antioxidant, resveratrol combats oxidative stress through multiple pathways, making it a promising therapeutic agent for AD.

Nrf2 serves as a critical regulator of cellular antioxidant responses. It regulates numerous genes, Such as NQO1, glutathione S-transferase (GST), γ-glutamylcysteine synthetase (GCS), xc−cystine/glutamate transporter (xCT), HO-1, and other small molecular substances that can reduce oxidative stress in cells [73,74]. The levels of Nrf2, HO-1, GSH and other antioxidants are decreased in the nuclei of neurons in the brains of patients with AD, Parkinson’s disease and other neurodegenerative diseases [75]. Meanwhile, the Nrf2-ARE pathway was weakened in APP/PS1 transgenic mice, while Nrf2 overexpression enhanced the activity of the Nrf2-ARE pathway to protect against Aβ toxicity and improve the spatial learning ability of APP/PS1 transgenic mice [76]. Resveratrol reverses Aβ-induced spatial memory impairment, iNOS elevation, lipid peroxidation, and HO-1 reduction in adult rats [77]. It also activates the Nrf2/HO-1 pathway, protecting against oxidative stress in AD models [78]. In addition, resveratrol protects astrocytes from GSH depletion and oxidative damage by increasing the levels of HO-1 [79]. Following Nrf2 activation, GSK-3β undergoes phosphorylation at tyrosine 216, enhancing its kinase activity. The activated GSK-3β then phosphorylates tyrosine kinase Fyn at specific threonine residues, enabling Fyn to phosphorylate Nrf2 at tyrosine 568. This post-translational modification triggers Nrf2 nuclear export and facilitates its SCF/β-TrCP-mediated proteasomal degradation [80]. RSV could activate the PI3K/AKT pathway and inhibit GSK-3β to promote Nrf2 nuclear translocation [81]. In addition, resveratrol may promote the nuclear accumulation of Nrf2 and subsequent activation of ARE-driven gene transcription through AMPK-mediated phosphorylation of Nrf2 at Ser550, coupled with the inhibition of GSK-3β [82,83].

### 2.3. Effect of Resveratrol on Energy Metabolism and Mitochondrial Homeostasis in AD

**Figure 3:**
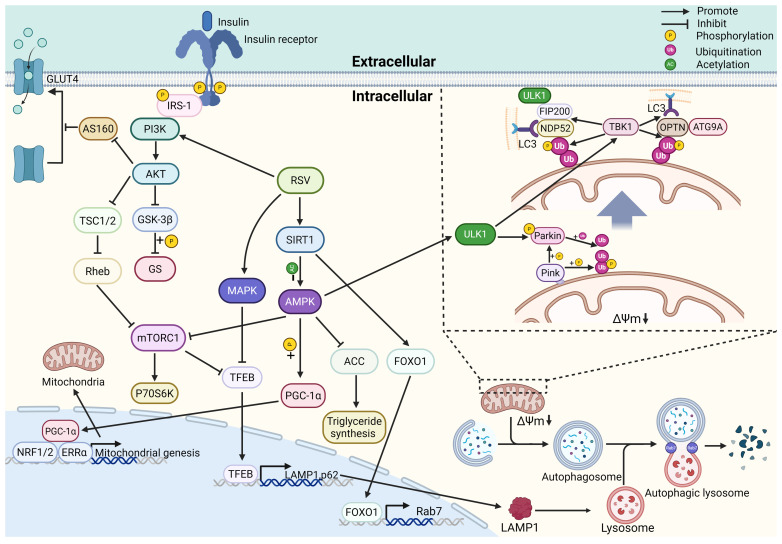
The mechanisms of resveratrol promote energy metabolism and mitochondrial homeostasis. (1) RSV enhances glucose uptake and glycogen synthesis by activating the PI3K/AKT pathway, thereby enhancing insulin sensitivity. (2) RSV reduces fatty acid synthesis and insulin resistance through AMPK/mTORC and ACC. (3) RSV promotes mitochondrial biogenesis through the SIRT1/AMPK/PGC-1α pathway. (4) RSV enhances mitophagy through the PINK1/Parkin pathway and facilitates autophagosome formation by activating ULK1. (5) RSV upregulates the expression of LAMP1 and p62 through the MAPK/TFEB and AMPK/mTORC1/TFEB pathways. (6) RSV promotes the formation of autolysosomes through the SIRT1/FOXO1/Rab7 pathway. PI3K, Phosphatidylinositol 3-Kinase; AKT, protein kinase B; AS160, TBC1 domain family member 4; GLUT4, glucose transporter type 4; IRS-1, insulin receptor substrate 1; TSC1/2, tuberous sclerosis complex 1/2; Rheb, ras homolog enriched in brain; mTORC1, mechanistic target of rapamycin complex 1; p70S6K, p70 ribosomal protein S6 kinase; GSK-3β, glycogen synthase kinase-3β; GS, glycogen synthase; RSV, resveratrol; SIRT1, sirtuin 1; AMPK, AMP-activated protein kinase; PGC-1α, peroxisome proliferator-activated receptor gamma coactivator 1-α; MAPK, mitogen-activated protein kinase; TFEB, transcription factor EB; FOXO1, forkhead box protein O1; Rab7, ras-associated binding protein 7; LAMP1, lysosomal-associated membrane protein 1; p62, sequestosome 1; NRF1/2, nuclear respiratory factor 1/2; ERRα, estrogen-related receptorα; ACC, acetyl-CoA carboxylase; ULK1, unc-51 like autophagy activating kinase 1; TBK1, TANK-binding kinase 1; FIP200, focal adhesion kinase family interacting protein of 200 kDa; NDP52, nuclear dot protein 52 kDa; OPTN, optineurin; ATG9A, autophagy related 9A; ΔΨm, mitochondrial membrane potential; LC3, microtubule-associated protein 1A/1B-light chain 3.

The brain accounts for an average of 2% of body weight but consumes 25% of total body glucose and 20% of total oxygen consumption. As one of the organs with high energy consumption, the brain is easily affected by impaired energy metabolism, and even slight changes in human brain energy metabolism are closely related to neurological dysfunction [84]. Glucose is the major substrate for energy metabolism in the adult brain under physiological conditions. Substantial evidence indicates that a significant reduction in glucose utilization is an early and consistent feature of AD, which actually occurs decades before clinical symptoms manifest [85]. Glucose metabolism is a multi-stage process that encompasses both cellular glucose uptake and its subsequent intracellular metabolic pathways. Disruptions in any of the steps will lead to disorders of energy metabolism. Indeed, both abnormal glucose transport due to insulin resistance and altered intracellular metabolism resulting from mitochondrial dysfunction have been well documented to occur in AD patients [86]. Here, we summarize how resveratrol preserves mitochondrial homeostasis and energizes mitochondrial metabolism, thereby slowing the progression of AD (Figure 3).

#### 2.3.1. Resveratrol Improves Energy Metabolism by Modulating Insulin-Related Pathways

Insulin and insulin-like growth factors are widely expressed in neurons and astrocytes and play important roles such as cell growth, metabolism, survival, gene expression and protein synthesis, neurotransmitter networks, and synaptic function. Numerous studies have shown that insulin resistance is closely related to the pathogenesis of AD and is a high-risk factor for AD [87]. Once bound to insulin, the insulin receptor (IR) changes its conformation and activates the endogenous tyrosine kinase activity of the β subunit to tyrosine phosphorylate itself and recruit insulin receptor substrates-1 (IRS-1). IRS-1 recruits and activates PI3K, which further activates AKT and inhibits AS160, thereby promoting the translocation of glucose transporter 4 (GLUT4), which in turn promotes cellular glucose uptake [88]. Decreased GLUT4 expression and impaired insulin signaling have been observed in the brains of postmortem AD patients [88,89]. Among them, the insulin signaling defect mainly involves the conduction defect of the PI3K/AKT pathway, which is more significant in type 2 diabetes mellitus and AD subjects [90]. Resveratrol can activate PI3K/AKT and AMPK to enhance GLUT4 expression and increase insulin sensitivity in peripheral tissues [91]. When insulin signaling is activated, AKT phosphorylates and inhibits GSK-3β and inhibits its activity, resulting in increased glycogen synthesis and decreased blood glucose [92]. In the brains of AD patients, the decreased phosphorylation of GSK-3β leads to insulin resistance and is closely related to tau protein hyperphosphorylation [93]. Resveratrol can increase GSK-3β phosphorylation and inhibit its activity via the PI3K-AKT pathway [81]. Notably, GSK-3β phosphorylation not only promotes glycogen synthesis (GS) to lower glucose but also inhibits tau hyperphosphorylation and promotes Nrf2 nuclear translocation, as previously discussed.

Rheb, a small GTPase localized on the lysosomal membrane, activates mTORC1 kinase activity directly in its GTP-bound state. The TSC1-TSC2 complex converts the active form of Rheb to its inactive GDP-bound state, generating negative regulation of mTORC1 [94]. AKT-mediated phosphorylation of TSC2 inhibits its GAP activity, leading to mTORC1 activation, which further leads to p70S6K phosphorylation activation [95]. p70S6K controls the expression of early adipogenic transcription factors, and activation of p70S6K would promote adipogenesis, a major risk factor for insulin resistance [96]. Resveratrol can activate AMPK phosphorylation, which in turn phosphorylates regulatory-associated protein of mTOR (Raptor), inhibiting mTORC1 activity and enhancing the effect of insulin signaling [32,97]. AMPK activation by resveratrol also increases insulin sensitivity by phosphorylating acetyl-CoA carboxylase (ACC), inhibiting triglyceride synthesis and increasing glucose uptake and fatty acid oxidation [98]. However, although resveratrol induced increases in ATP and GTP in a dose-dependent manner, it resulted in decreased glucose consumption and lactate release, suggesting that it may inhibit energy-wasting processes rather than enhance energy production [99]. Resveratrol may also act as a caloric restriction mimetic, possibly by activating SIRT1, significantly increasing insulin sensitivity and reducing insulin-like growth factor-1 (IGF-I) levels, thereby protecting against insulin insensitivity induced by high-calorie diets in neurodegenerative diseases [100]. Consistent with these findings, resveratrol has neuroprotective effects on diabetic and AD comorbidity rat models by activating SIRT1 signaling [101].

#### 2.3.2. Resveratrol Promotes Mitochondrial Homeostasis by Modulating Mitochondrial Biogenesis and Mitophagy

In general, neurons are powered by aerobic oxidation of glucose, so mitochondria are essential for neuronal energy metabolism, and their dysfunction will impair neuronal function [102]. Genes involved in glycolysis, tricarboxylic acid (TCA) cycle and oxidative phosphorylation are down-regulated in AD patients, and mitochondrial electron transport chain activity is generally inhibited [103]. Currently, extensive research suggests that mitochondria may mediate, drive, or contribute to multiple AD pathologies, and mitochondrial dysfunction in AD is a reasonable therapeutic target [104]. Mitochondrial quantity and quality are tightly regulated by two basic and opposing mechanisms, mitochondrial biogenesis and mitophagy, which are tightly coordinated in response to cellular energy demands and environmental changes [105]. Studies have shown that increasing mitochondrial protein homeostasis by increasing mitochondrial translation and mitophagy can reduce the toxicity and accumulation of Aβ and attenuate disease progression [106]. Therefore, increasing the generation of newborn mitochondria and aging damage mitophagy clearance to increase the mitochondrial turnover cycle and maintain mitochondrial homeostasis are promising therapeutic approaches for AD.

##### Mitochondrial Genesis

Peroxisome proliferator-activated receptor-γ coactivator1-α (PGC-1α) is a transcriptional coregulator that regulates mitochondrial biogenesis by interacting with transcription factors nuclear respiratory factor 1 (NRF1), nuclear respiratory factor 2 (NRF2) and estrogen-related receptor α (ERRα) [107]. NRF1 and NRF2 can bind to the promoters of mitochondrial transcription factor A (TFAM), mitochondrial transcription factor B1 (TFB1M) and mitochondrial transcription factor B2 (TFB2M) genes, and their protein products serve as key regulators that directly govern and stimulate mitochondrial DNA (mtDNA) transcription. ERRα binds to PGC-1α to promote mitochondrial oxidative phosphorylation (OXPHOS) and the expression of mitochondrial genes [108]. AMP-activated protein kinase (AMPK) induces mitochondrial biogenesis through direct phosphorylation of PGC-1α at THR 177 and Ser 538. SIRT1 can also directly deacetylate PGC-1α to increase PGC-1α activity [109]. The levels of PGC-1α and SIRT1 were significantly decreased in the brains of AD patients, indicating mitochondrial dysfunction. Resveratrol can activate AMPK and SIRT1, increase the content and deacetylation of PGC-1α, and further increase mitochondrial biogenesis and improve mitochondrial quality.

##### Mitochondrial Autophagy

Mitophagy, a selective form of macroautophagy, involves the encapsulation of damaged or superfluous mitochondria within double-membraned autophagosomes, which further fuse with lysosomes for degradation. Ubiquitin-mediated mitophagy is essential for the dynamic regulation of mitochondria. Under steady-state conditions, PTEN-induced putative kinase 1 (PINK1) is transported to the mitochondrial membrane space for proteolytic cleavage, and the cleaved PINK1 is transported to the cytoplasm for proteasomal degradation [110]. However, when the mitochondrial membrane potential (ΔΨm) was depolarized, the import of PINK1 was blocked, which stabilized PINK1 on the OMM and resulted in the recruitment of Parkin to the damaged mitochondria [111]. Parkin, an E3 ubiquitin ligase in the cytosol, can ubiquitinate mitochondria, and its activity is enhanced by PINK1-mediated phosphorylation at Ser65. PINK1 also phosphorylates ubiquitin at Ser65, further regulating Parkin activity [112]. Meanwhile, the autophagy receptor proteins optineurin (OPTN), CALCOCO2 (NDP52), p62/SQSTM1, NBR1 and TAX1BP1 were recruited to the ubiquitylated mitochondria. Notably, although all five autophagic junctions are recruited to damaged mitochondria during mitophagy, only OPTN is essential for mitochondrial clearance [113]. Subsequently, LC3 (microtubule-associated protein 1A/1B-light chain 3) binds to the LC3-interacting region (LIR) domain of autophagy receptor proteins to form autophagic vesicles. Formation of autophagosome membranes around ubiquitinated mitochondria depends on the binding of NDP52 and OPTN to the core autophagy proteins FIP200 and ATG9A, respectively [114]. Phosphorylation of OPTN by TANK-binding kinase 1 (TBK1) enhanced its binding to LC3 and Ub chains [113,115], while phosphorylation of NDP52 by TBK1 enhanced the interaction with the FIP200/ULK1 complex, though this effect was not essential [116].

Hippocampal neurons in AD samples showed altered mitochondrial morphology, mainly in terms of reduced size and excessive mitochondrial damage, compared to healthy controls. The basal level of mitophagy in the hippocampus of AD patients is 30 to 50% lower than normal, and damaged mitochondria accumulate. The mitophagy initiation proteins *p*-TBK1 (Ser172) and *p*-ULK1 (Ser555) were inactivated in all AD individuals [117]. Resveratrol activates TBK1 and phosphorylates ULK1 at Ser555 via AMPK activation [118,119]. ULK1 also phosphorylates Parkin at Ser108 in an AMPK-dependent manner when mitochondria are under stress, which is required for PINK1-mediated phosphorylation and activation of Parkin at Ser65 and Parkin-mediated mitochondrial clearance [120]. Additionally, ULK1 directly phosphorylates mitophagy receptors, such as FUNDC1, BNIP3, NIX and BCL2L13, to promote mitophagy receptor-mediated mitophagy [121]. Thus, resveratrol-induced ULK1 activation via AMPK is crucial for eliminating impaired mitochondria in AD patients. In addition, resveratrol can also regulate mitochondrial quality control and reduce inflammatory response and oxidative stress through the NRF1/2 pathway and the Pink1/Parkin pathway [122,123].

##### Lysosomal Acidification and the Formation of Autophagy Lysosomes

Abnormal accumulation of autophagic vacuoles is a typical feature of neurons from AD patients, which may be caused by defective lysosomal function and dysfunctional fusion between autophagosomes and lysosomes [124]. ERK in the MAPK pathway phosphorylates S142 of TFEB and inhibits its nuclear translocation to regulate lysosome biogenesis [125]. The inhibitory effect of resveratrol on MAPK may also increase the nuclear translocation of TFEB to coordinate lysosome biogenesis and autophagy. Resveratrol also increases TFEB transcriptional activity by activating SIRT1 and deacetylating TFEB at K116, thereby upregulating the transcription of genes (LAMP1, p62) related to autophagy and lysosomes [126]. TFEB serves as a master transcriptional regulator of autophagy and lysosome biogenesis, whose activity is suppressed by MTORC1-mediated phosphorylation. Moreover, AMPK enhanced the transcriptional activity of TFEB and TFE3 due to its mTORC1 inhibition [127]. As an activator of AMPK, resveratrol enhances autophagy, at least in part, through AMPK/mTORC1-mediated augmentation of TFEB and TFE3 transcriptional activity. Rab7, a small GTPase, regulates the fusion of autophagosomes with lysosomes during the late stages of autophagy and is essential for sustaining autophagic flux. Resveratrol can increase Rab7 expression by activating SIRT1 to deacetylate Foxo1, effectively alleviating autophagy dysfunction [66,128].

### 2.4. Effect of Resveratrol on Neuroprotective and Regenerative Effects in AD

**Figure 4:**
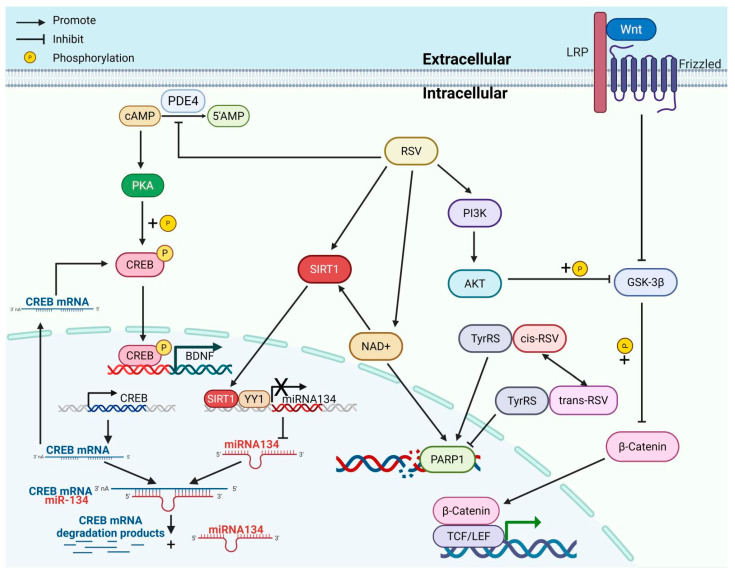
The mechanisms of resveratrol promote neuroprotective and regenerative effects. (1) RSV enhances the production of BDNF through SIRT1/miRNA144/CREB/BDNF and PKA/CREB/BDNF pathways. (2) RSV promotes DNA damage repair via the PARP1 pathway while mitigating the inhibitory effect of NAD+ depletion on SIRT1 activity. (3) cis-RSV facilitates PARP1-mediated DNA repair by binding to TyrRS and mimicking a “tyrosine-free” conformation, whereas trans-RSV inhibits the TyrRS/PARP1 function by simulating a “tyrosine-bound” conformation. (4) RSV promotes adult hippocampal neurogenesis through the Wnt/β-catenin and PI3K/AKT/GSK-3β/β-catenin signaling pathways. cAMP, cyclic adenosine monophosphate; PDE4, phosphodiesterase 4; 5′AMP, 5′-adenosine monophosphate; PKA, protein kinase A; CREB, cAMP response element-binding protein; BDNF, brain-derived neurotrophic factor; SIRT1, sirtuin 1; YY1, Yin Yang 1; RSV, resveratrol; AMPK, AMP-activated protein kinase; NAD+, nicotinamide adenine dinucleotide; PARP1, Poly(ADP-ribose) Polymerase 1; cis-RSV, cis-resveratrol; trans-RSV, trans-resveratrol; TyrRS, tyrosyl-tRNA synthetase; PI3K, phosphatidylinositol 3-kinase; AKT, protein kinase B; LRP, low-density lipoprotein receptor-related protein; GSK-3β, glycogen synthase kinase-3β.

Resveratrol activates the SIRT1 pathway to attenuate neuronal damage, suppress apoptosis, promote neural stem cell proliferation and differentiation, enhance axonal regeneration, and improve cognitive and motor functions, offering a novel strategy for AD (Figure 4).

#### 2.4.1. Resveratrol Exerts Neuroprotective Effects by Promoting the Production of BDNF

SIRT1 can form a transcriptional repressor complex with Yin Yang 1 (YY1) to inhibit the expression of miR-134. Overexpression of miR-134 binds to the mRNA of cAMP response element-binding protein (CREB), which significantly reduces the expression of CREB. This reduction further down-regulates the expression of brain-derived neurotrophic factor (BDNF), thereby impairing synaptic plasticity and memory formation [129]. Resveratrol prevents the Aβ-induced reduction in SIRT1 expression and CREB phosphorylation in the rat hippocampus. Additionally, it upregulates the expression of BDNF and ameliorates the spatial learning deficits [130,131]. Resveratrol also increases intracellular cAMP content by inhibiting phosphodiesterase 4 (PDE4) activity, which further activates the downstream PKA/CREB/BDNF pathway to improve memory performance [132]. Meanwhile, resveratrol also increases the expression of anti-apoptotic factor BCl-2 to reduce cell apoptosis [132,133].

#### 2.4.2. Resveratrol Exerts Neuroprotective Effects by Modulating PARP1 Activity

When DNA damage occurs, poly (ADP-ribose) polymerase 1 (PARP1) is activated to promote DNA repair, but it leads to the loss of NAD+ and acetyl-coa, which further leads to the inhibition of SIRT1 [134]. In AD, possibly due to the inability of damaged mitochondria to be cleared, a significant quantity of ROS is generated. Excessive ROS puts neurons in oxidative stress, leading to nuclear DNA damage, showing persistent PARP1 over-activation, reduced SIRT1 activity, and NAD+ depletion [135]. Although short-term activation of PARP1 is beneficial for genome stability because it promotes DNA repair; However, persistent activation of PARP1 may contribute to pathology in age-related diseases. Resveratrol can enhance the activity of SIRT1 by regulating NAD+ production and reducing the negative effects of PARP1 activation on the body [134,136]. In addition, tyrosine inhibits tyrosyl-tRNA synthetase (TyrRS)-mediated activation of PARP1. Increased tyrosine levels in the AD brain consume TyrRS, reducing histone serine-ADP-ribosylation, and ultimately leading to neuronal DNA damage [137]. Cis-resveratrol (cis-RSV) interacts with TyrRS, inducing a conformation that mimics the tyrosine-free state. This binding upregulates TyrRS activity, subsequently enhancing histone serine-ADP-ribosylation-mediated DNA repair mechanisms in a TyrRS-dependent manner. However, the retention of trans-resveratrol (trans-RSV) at the active site of TyrRS mimics its tyrosine binding conformation, inhibiting TyrRS/PARP1-mediated protective stress responses, and inducing neurodegeneration in rat cortical neurons [137]. Notably, PARP1 activity is enhanced in the brain of AD patients, and Aβ can also enhance PARP1 activity, which seems to be an early and important event in the pathogenesis of AD [138]. From this point of view, the inhibitory effect of trans-resveratrol on PARP1 over-activation also seems to have a beneficial side. Emerging evidence indicates that trans-resveratrol and its metabolites can undergo isomerization to cis-resveratrol under physiological conditions [139]. These studies further demonstrate the dual role of resveratrol in AD neuroprotection.

#### 2.4.3. Resveratrol Promotes Adult Hippocampal Neurogenesis

Adult hippocampal neurogenesis (AHN) persists throughout human life, and these newly generated neurons are continuously integrated into the neural circuit of the brain [140]. However, AHN is significantly reduced in AD patients and AD transgenic mouse models, which may be the root cause of progressive neuronal loss in AD patients [141]. Resveratrol increased hippocampal neurogenesis and reduced astrocyte hypertrophy and microglia activation [142]. Resveratrol also reduced the inhibition of neurogenesis by early postpartum ethanol exposure and increased the density and maturity of dendritic spines [143]. In the chronic fatigue mouse model, resveratrol alleviates fatigue and hippocampal atrophy by inhibiting apoptosis and promoting neurogenesis [144]. Resveratrol protects human neural stem cells (hNSC) from amyloid-induced inflammation and oxidative stress in an AMPK-dependent manner [83]. However, some studies report that resveratrol impairs hippocampal-dependent spatial learning and memory by activating AMPK to reduce neural progenitor cell (NPC) proliferation and survival in the dentate gyrus [145].

Accumulating evidence suggests that Wnt signaling regulates multiple aspects of adult hippocampal neurogenesis. It has been found that Wnt signaling activity is decreased in the brain of AD patients, and there is a correlation between Aβ-induced neurotoxicity and reduced cytoplasmic level of β-catenin [146]. Aβ also binds to the extracellular cysteine-rich domain of Frizzled to inhibit Wnt/β-catenin signaling [147]. Wnt signaling inhibition is associated with memory loss, tau phosphorylation, and Aβ formation and aggregation in the AD transgenic mouse model, further suggesting that Wnt signaling dysfunction accelerates the pathogenesis of AD [148]. Resveratrol (RSV) has been shown to enhance β-catenin nuclear translocation by activating SIRT1, which reduces the acetylation level of β-catenin in cells [149]. Moreover, RSV upregulates the expression of Wnt and β-catenin, thereby activating the Wnt/β-catenin signaling pathway and promoting axonal regeneration following spinal cord injury (SCI) [150]. Additionally, resveratrol may activate the Wnt/β-catenin signaling pathway via the PI3K/AKT/GSK-3β axis, facilitating the proliferation and differentiation of neural stem cells [151]. It is noteworthy that resveratrol also has a concentration-dependent effect. At 10 μM concentration, it promotes cell differentiation through activation of the Wnt/β-catenin signaling pathway, while at 20 μM concentration, it has the opposite effect [152,153].

## 3. Strategy Optimization of Resveratrol in the Treatment of AD

### 3.1. Combination Therapies

Combining resveratrol with other therapeutic agents has shown superior efficacy compared to resveratrol monotherapy. For instance, the combination of resveratrol and donepezil showed better clearance of β-amyloid plaques and NFTs, lower levels of malondialdehyde and oxidative stress, and less activated microglia [154]. Pretreatment of rat brain cultures with a combination of ethanol and resveratrol at low protective concentrations enhances neuroprotective effects and inhibits neuroapoptosis by synergistically upregulating the NMDDAR-PKC-Prx2 pathway and other antioxidant proteins [155]. The combination of 1,25-dihydroxyvitamin D3 and resveratrol ameliorates neuronal degeneration by synergistically modulating endoplasmic reticulum (ER) stress, insulin signaling and tau hyperphosphorylation in SH-SY5Y cells [156]. Melatonin(MEL) and resveratrol synergistically activate the PI3K-AKT pathway, inhibiting the ubiquitin-dependent proteasomal degradation of HO-1, thereby enhancing the neuroprotective effect of RSV against oxidative damage [157]. Notably, the combined treatment of MEL and RSV does not show any significant synergistic effect on the recovery of GSH depletion or the inhibition of GSK-3β activation induced by Aβ(1–42), but instead inhibited AMPK phosphorylation [158].

### 3.2. Resveratrol Derivatives

Structural modifications of resveratrol have been explored to improve its bioavailability and therapeutic efficacy. Hydroxylation of the 4- and 4′-positions in the trans-stilbene structure enhances the water solubility and functional versatility of the parent resveratrol molecule. For instance, polyhydroxylated RSV derivatives containing fewer than three hydroxyl groups exhibit lower oral bioavailability [159]. Trans 4,4′-dihydroxyastragalus (DHS), when completely dissolved by hydroxypropyl-β-cyclodextrin, shows a higher bioavailability than resveratrol due to its rapid uptake [160]. Piceatannol (3′,4′,3,5-tetrahydroxytrans stilbene) is another hydroxylated derivative of resveratrol and has a more complex metabolite than resveratrol. Although the area under the curve (AUC) of plasma concentration of piceatannol was smaller than that of resveratrol, it was significantly increased when it was combined with resveratrol, indicating that its metabolic stability was increased [161]. Piceatannol has a stronger neuroprotective effect than resveratrol, which can more effectively block the accumulation of intracellular ROS and cell apoptosis induced by Aβ treatment in PC12 cells [162]. Piceatannol (but not resveratrol) attenuated 4-hydroxynonenal-induced apoptosis in PC12 cells by blocking the activation of *c*-Jun *N*-terminal kinase [163]. Pterostilbene, the methoxylated derivative of resveratrol, exhibits higher lipophilicity and bioavailability than resveratrol. Pterostilbeni can up-regulate the levels of SIRT1, Nrf2 and SOD, and inhibit the mitochondria-dependent apoptosis induced by Aβ [164]. Studies have shown that pterostilbene has a stronger neuroprotective effect and a more effective role in the prevention and treatment of AD compared with resveratrol [165]. Polydatin, a glycosylated derivative of resveratrol, has higher bioavailability. Although both resveratrol and polydatin have strong anti-oxidative stress ability, polydatin promotes the activities of total superoxide dismutase (T-SOD) and catalase (CAT) in plasma and increases the content of glutathione, which is superior to resveratrol [166].

### 3.3. Advanced Drug Delivery Systems

Nanotechnology plays a pivotal role in drug delivery in the central nervous system, which can increase the stability and solubility of drugs. By combining the unique Aβ uptake properties of SeNPs with the natural antioxidant RSV to form RSV@SeNPs, which significantly protected PC12 cells from Aβ(1–42)-Cu^2+^ complex-induced cell death [167]. Resveratrol-selenium nanoparticles (RSV-SeNPs) minimize neuroinflammation and neurotoxicity in a rat model of AD by regulating SIRT1/miRNA-134 and PI3K/AKT/GSK-3β pathways, maximizing the therapeutic potential of RSV for AD [168]. Oral resveratric-selenium-peptide nanocomposites can alleviate AD-like pathological changes by inhibiting the deposition of Aβ and inducing ROS and inflammatory factors. It also increases antioxidant enzyme activity and alleviates intestinal microbiota disorder, especially oxidative stress and inflammation-related bacteria [169]. By combining 3-hydroxypyridine-4-one complex with resveratrol and Aβ, researchers have designed and synthesized deferiprone-resveratrol hybrids. It has good inhibitory activity against Aβ(1–42) aggregation, excellent antioxidant activity, and effective metal chelation ability [170]. Resveratrol-loaded oral bilosomes are superior to conventional drug suspensions in reducing oxidative stress, inflammation, Aβ and tau pathology, as well as improving behavioral outcomes in animal models [171]. Brain-targeted peptide-modified resveratrol nanoparticles (TG-RSV-CS/TPP-NPs) enable precise delivery of resveratrol to the brain. This system has been shown to ameliorate cognitive impairment of obesity-related AD mice and reduce the levels of phosphorylated tau and Aβ aggregation through the JNK/AKT/GSK-3β pathway. Due to the presence of brain-targeting peptides, TG-RSV-CS/TPP-NPs can better target the brain to regulate brain glucose homeostasis, oxidative stress and neuroinflammation [172].

### 3.4. Intranasal Delivery

The intranasal route of administration is an important alternative to systemic (oral and parenteral) administration to the brain and plays an important role in the treatment of central nervous system diseases. In this way, drugs can be delivered directly to the brain via the olfactory pathway, and the presence of drugs in the olfactory bulb in turn increases drug bioavailability in the brain, reducing drug degradation and wastage through the body. Compared with oral resveratrol suspension, intranasal resveratrol treatment significantly improved the memory performance in scopolamine-induced amnestic rats [173]. Intranasal gelated liquid crystals containing resveratrol can improve learning and memory in AD models while alleviating neuroinflammation [174]. Resveratrol and superparamagnetic iron oxide nanoparticles (SPION) were encapsulated into chitosan-coated bile bodies and incorporated into alginate/PVP wafers for sustained intranasal delivery. Compared with conventional bile body and resveratrol suspension, it significantly improved cognitive and memory functions, reduced the levels of inflammatory markers and the expression levels of NF-κB and P38 in AD mice [175].

### 3.5. Lipid-Based Nanoparticles

The delivery system of lipid nanoparticles, which can penetrate the BBB, shows excellent potential to improve the bioavailability of resveratrol. Researchers have recently developed two novel lipid nanoparticle-based resveratrol nanodelivery systems that provide protection for incorporated resveratrol and allow controlled release after uptake, significantly enhancing the oral bioavailability and stability of resveratrol [176]. Compared with free resveratrol, resveratrol-loaded lipid-core nanocapsules (RSV-LNC) can significantly increase the concentration of resveratrol in brain tissue and greatly improve the safety of the intestinal tract [177]. RSV-LNC was superior to free resveratrol in protecting against Aβ1-42-induced abnormal overactivation of *c*-Jun *N*-terminal kinase (JNK) and GSK-3β, as well as in protecting against neuroinflammation, oxidative stress and neuroprotection. Resveratrol-loaded solid lipid nanoparticles (R-SLN) resulted in 4.5-fold higher levels of resveratrol in the brain compared with free resveratrol administration. It also activated the Nrf2/HO-1 pathway more effectively to alleviate mitochondrial oxidative stress in vascular dementia [178]. Studies have shown that long-term treatment with resveratrol may inhibit cells by inducing ROS production and activation of caspase-3. The use of resveratrol-loaded polymeric micelles can not only protect PC-12 cells from Aβ-induced damage, but also reduce the toxic effect of long-term use of resveratrol on cells [179].

### 3.6. Targeted Crosslinking

The brain targeting and bioavailability of resveratrol can be greatly improved by cross-linking specific molecules on solid lipid nanoparticles. For example, by loading apolipoprotein onto solid lipid nanoparticles loaded with resveratrol, the permeability of resveratrol was greatly increased by binding apolipoprotein E to the overexpressed LDL receptor 1 at the BBB [180]. APOE-modified resveratrol-loaded liposomes increased the permeability of resveratrol in the BBB and the uptake of resveratrol in bEnd.3 and N2a cells, and showed better effects in reducing energy oxidative stress and neuroinflammation in the brain of APP/PS-1 mice [181]. Loading rabies virus glycoprotein (RVG29) and triphenylphosphine cationic (TPP) molecules attached to the surface of erythrocyte membrane (RVG/TPP NP@RBCm) onto nanostructured lipid carriers (NLC) not only facilitates resveratrol to cross the BBB, but also further targets neuronal cells to further localize to mitochondria. This can achieve sustained and stable drug release and improve biocompatibility to alleviate Aβ-related mitochondrial oxidative stress and relieve AD symptoms [182]. Neuronal mitochondrion-targeting micelles (CT-NM) were prepared by using brain neuron-specific binding neural cell adhesion molecule (NCAM) mimetic peptide C3 and triphenyl phosphate (TPP) for mitochondrial targeting. It significantly increased the concentration of resveratrol in neuronal mitochondria and restored the cognitive performance of APP/PS1 transgenic mice to the level of wild-type mice through various mechanisms, reflecting its great therapeutic potential [183].

## 4. Conclusions and Future Perspectives

**Figure 5:**
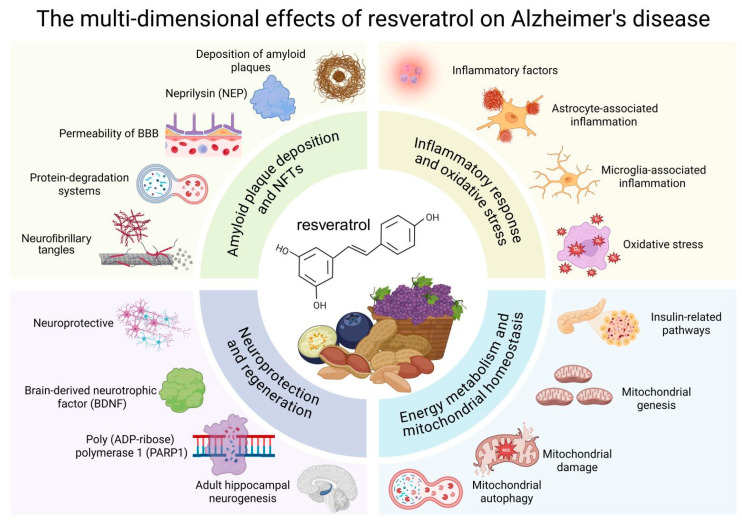
The therapeutic effects of RSV on AD are manifested in the following four aspects: (1) amyloid plaque deposition and NFTs, (2) inflammatory response and oxidative stress, (3) energy metabolism and mitochondrial homeostasis, and (4) neuroprotection and regeneration. BBB, Blood–Brain Barrier.

With the increase in people’s average life span, the incidence of neurodegenerative diseases, particularly AD, has risen significantly, posing substantial social and economic challenges. Resveratrol, a natural small-molecule compound, has emerged as a promising therapeutic agent for the treatment of various diseases. Extensive research has demonstrated that resveratrol alleviates AD symptoms and mitigates disease progression through multiple mechanisms, with minimal side effects. In this review, we comprehensively examine the role of resveratrol in AD, focusing on four key aspects: (1) amyloid plaque deposition and NFTs, (2) inflammatory response and oxidative stress, (3) energy metabolism and mitochondrial homeostasis, and (4) neuroprotection and regeneration (Figure 5). In addition, resveratrol suffers from poor water solubility, suboptimal in vivo stability, and a pronounced first-pass effect, which severely limit its pharmaceutical development and clinical application. Future research must prioritize the development of safe novel derivatives or advanced delivery systems to comprehensively enhance its therapeutic efficacy.

## Data Availability

The original contributions presented in this study are included in the article. Further inquiries can be directed to the corresponding authors.

## Abbreviations

The following abbreviations are used in this manuscript:

AD	Alzheimer’s disease
ACC	Acetyl-CoA carboxylase
AHN	Adult hippocampal neurogenesis
ALP	Autophagy-lysosomal pathway
AMPK	AMP-activated protein kinase
Aph-1	Anterior pharynx defective-1
APP	Amyloid precursor protein
ARE	Antioxidant response elements
ASC	Apoptosis-associated speck-like protein containing a CARD
ATG5	Autophagy-related protein 5
AUC	Area under the curve
Aβ	Amyloid β-protein
BACE1	β-site APP cleavage enzyme
BBB	Blood–brain barrier
BDNF	Brain-derived neurotrophic factor
Ca^2+^/CaMKKβ	Calcium/calmodulin-dependent protein kinase kinase-β
CALCOCO2/NDP52	Nuclear dot protein 52
CAT	Catalase
CCL2	Chemokine ligand 2
COX-2	Cyclooxygenase-2
CREB	cAMP response element-binding protein
Cul3	Cullin 3
DHS	4,4′-dihydroxyastragalus
DPN	Diabetic peripheral neuropathy
ERRα	Estrogen-related receptor α
fAβ42	fibril Aβ42
FGF-2	Fibroblast growth factor-2
GABARAPL1	Gamma-aminobutyric acid receptor-associated protein-like 1
GCN2	General control nonderepressible 2
GCS	γ-glutamylcysteine synthetase
GFAP	Glial fibrillary acidic protein
GLUT4	Glucose transporter 4
GSDMD	Gasdermin D
GSH	Glutathione
GSK-3β	Glycogen synthase kinase
GST	Glutathione S-transferase
hNSC	Human neural stem cells
HO-1	Heme oxygenase-1
IFN-γ	Interferon-γ
IGF-I	Insulin-like growth factor-1
IL-1β	Interleukin-1β
IL-4	Interleukin-4
iNOS	Inducible NO synthase
IR	Insulin receptor
JNK	Jun N-terminal kinase
Keap1	Kelch-like ECH-associated protein 1
LIR	LC3-interacting region
LKB1	Liver kinase B1
LRP1	Lipoprotein receptor-associated protein 1
MAPK	Mitogen-activated protein kinase
MCP-1	Monocyte chemoattractant protein-1
MDC	Macrophage-derived chemokine
MEL	Melatonin
MMP-9	Matrix metalloproteinase-9
mTOR	Mechanistic target of rapamycin
mTORC1	mTOR complex 1
MyD88	Primary response gene 88
NFTs	Neurofibrillary tangles
NF-κB	Nuclear Factor kappa-B
NMDA	N-Methyl-D-Aspartate
NO	Nitric oxide
NPC	Neural progenitor cell
NRF1/2	Nuclear respiratory factor 1/2
Nrf2/NFE2L2	Nuclear factor erythroid 2-related factor 2
PARP1	Poly (ADP-ribose) polymerase 1
PDE	Phosphodiesterase
PDE4	Phosphodiesterase 4
Pen-2	Presenilin enhancer-2
PGC-1α	Peroxisome proliferator-activated receptor-γ coactivator1-α
PGE2	Prostaglandin E2
PHFs	Paired helical filaments
PI3K	Phosphatidylinositol 3-kinase
PINK1	PTEN-induced putative kinase 1
PP2A	Protein phosphatase 2A
PSEN1	Presenilin1
RAGE	Receptor for advanced glycation end
ROS	Reactive oxygen species
RSV	Resveratrol
sAPPβ	Soluble APP beta protein
SIRT1	Sirtuin 1
sLRP1	Soluble form of LRP1
sMafs	small Maf proteins
SOCS1	Suppressor of cytokine signaling 1
SP	Senile plaques
SPION	Superparamagnetic iron oxide nanoparticles
SQSTM1/p62	Sequestosome 1
STAT1	Activator of transcription 1
TBK1	TANK-binding kinase 1
TCA	Tricarboxylic acid
TFAM	Promoters of mitochondrial transcription factor A
TFB1M	Mitochondrial transcription factor B1
TFB2M	Mitochondrial transcription factor B2
TLR4	Toll-like receptor 4
TNF-α	Tumor necrosis factor-α
TRX	Thioredoxin
TTR	Transthyretin
TXNIP	Thioredoxin-interacting protein
ULK1	Serine/threonine-protein kinase 1
UPS	Ubiquitin-proteasome system
xCT	Xc−cystine/glutamate transporter
YY1	Yin Yang 1
